# One-stop interventional procedure for bicuspid aortic stenosis in a patient with coexisting aortic coarctation: a case report

**DOI:** 10.3389/fcvm.2023.1162203

**Published:** 2023-05-04

**Authors:** Xingwei He, Menaka Dhuromsingh, Wanjun Liu, Qiang Zhou, Hesong Zeng

**Affiliations:** ^1^ Division of Cardiology, Department of Internal Medicine, Tongji Hospital, Tongji Medical College, Huazhong University of Science and Technology, Wuhan, China; ^2^ Hubei Provincial Engineering Research Center of Vascular Interventional Therapy, Wuhan, China; ^3^ Department of General Medicine, Sir Seewoosagur Ramgoolam National Hospital, Pamplemousses, Mauritius

**Keywords:** aortic coarctation, one'stop, case report, interventional procedure, bicuspid aortic valve

## Abstract

**Introduction:**

Coarctation of the aorta (CoA) is usually diagnosed and corrected early in life. Most untreated patients with CoA usually die before 50 years of age. Adult patients with concomitant CoA and severe bicuspid aortic stenosis are relatively rare and present complex management challenges without standard guidelines.

**Case summary:**

A 63-year-old female patient with uncontrolled hypertension was admitted due to chest pain and dyspnea upon exertion (NYHA grades III). Echocardiogram showed a severely calcified and stenotic bicuspid aortic valve (BAV). A severe stenotic calcified eccentric aortic coarctation 20 mm distal to the left subclavian artery (LSA) was discovered by computed tomography (CT) angiography. Following consultation with the cardiac team and patient willingness, we performed a one-stop interventional procedure to repair both defects. First, a cheatham-platinum (CP) stent was implanted *via* the right femoral access, immediately distal to the LSA. Due to the markedly twisted and angled descending aortic arch, we chose to perform transcatheter aortic valve replacement (TAVR) *via* the left common carotid artery. The patient was discharged and followed up for 1 year without symptoms.

**Discussion:**

Although surgery is still the main treatment for these diseases, it is not suitable for high-risk surgical patient. Transcatheter intervention for patients with severe aortic stenosis complicated with CoA simultaneously is rarely reported. The success of this procedure depends on the patient's vascular condition, the skills of the heart team, and the availability of the technical platform.

**Conclusion:**

Our case report demonstrates the feasibility and efficacy of a one-stop interventional procedure in an adult patient with concurrent severely calcified BAV and CoA *via* two different vascular approaches. Transcatheter intervention, in contrast to traditional surgical approaches or two-stop interventional procedures, as a minimally invasive and novel method, offers a wider range of therapeutic methods for such diseases.

## Introduction

Coarctation of the aorta (CoA) is a common defect that accounts for 5%–8% of all congenital heart defects (1, 2). CoA is often associated with concomitant cardiac pathologies, particularly bicuspid aortic valve (BAV), mitral valve abnormality, and ventricular septal defect (3). It has been reported that BAV is present in approximately two-thirds of CoA patients and has been linked to an increased risk for valvular dysfunction and aortic dilation. Most untreated patients with CoA usually die before 50 years of age (4). Adult patients with concomitant CoA and severe bicuspid aortic stenosis are relatively rare and present complex management challenges without standard guidelines (5, 6). Here, we present a case of a one-stop interventional procedure using two different vascular approaches in a patient with CoA and severe bicuspid aortic stenosis.

## Case presentation

A 63-year-old female patient was admitted for chest pain, dyspnea on exertion in the previous 8 months, and aggravation for 1 week (NYHA grades III). She reported uncontrolled hypertension despite taking antihypertensive medications such as amlodipine 5 mg and valsartan 80 mg once daily. The physical examination showed a blood pressure of 160/110 mmHg, a rhythmic pulse of 83 beats/min, and a harsh grade 4/6 systolic ejection murmur in the right second intercostal space, which led to the carotids. A blood pressure difference of 10mmHg was observed in both lower extremities when compared to that of an arm. The blood pressure of both lower limbs is 10 mmHg lower than the arm. There were no other significant clinical findings and family history of cardiovascular disease. Blood tests showed elevated N-terminal pro brain natriuretic peptide (NT-proBNP) levels of 2,580 pg/ml (normal range 300–900 pg/ml). There were no significant abnormalities in routine blood test, liver and kidney function, and Troponin I.

**Figure 1:**
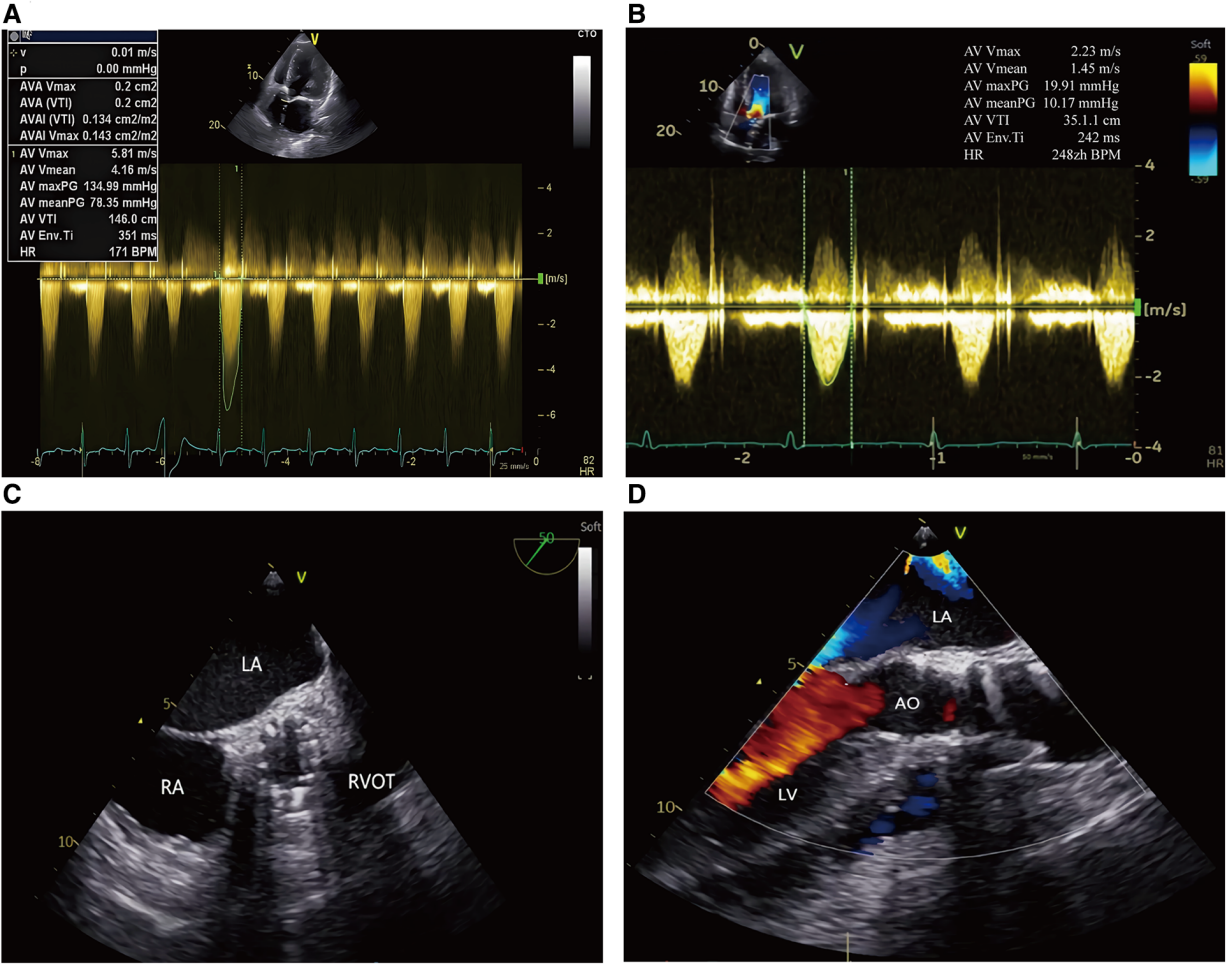
Trans-esophageal echocardiography in TAVR procedure. (**A**, **B**) Continuous Doppler showing the peak transvalvular pressure gradient: pre and post TAVR; (**C**) Postoperative echocardiography showed the prosthetic aortic valve with good morphology; (**D**) Postoperative echocardiography showed no significant aortic regurgitation after TAVR. LA, left atrium; RA, right atrium; LV, left ventricle; RVOT, right ventricular outflow tract; AO, aorta.

**Figure 2:**
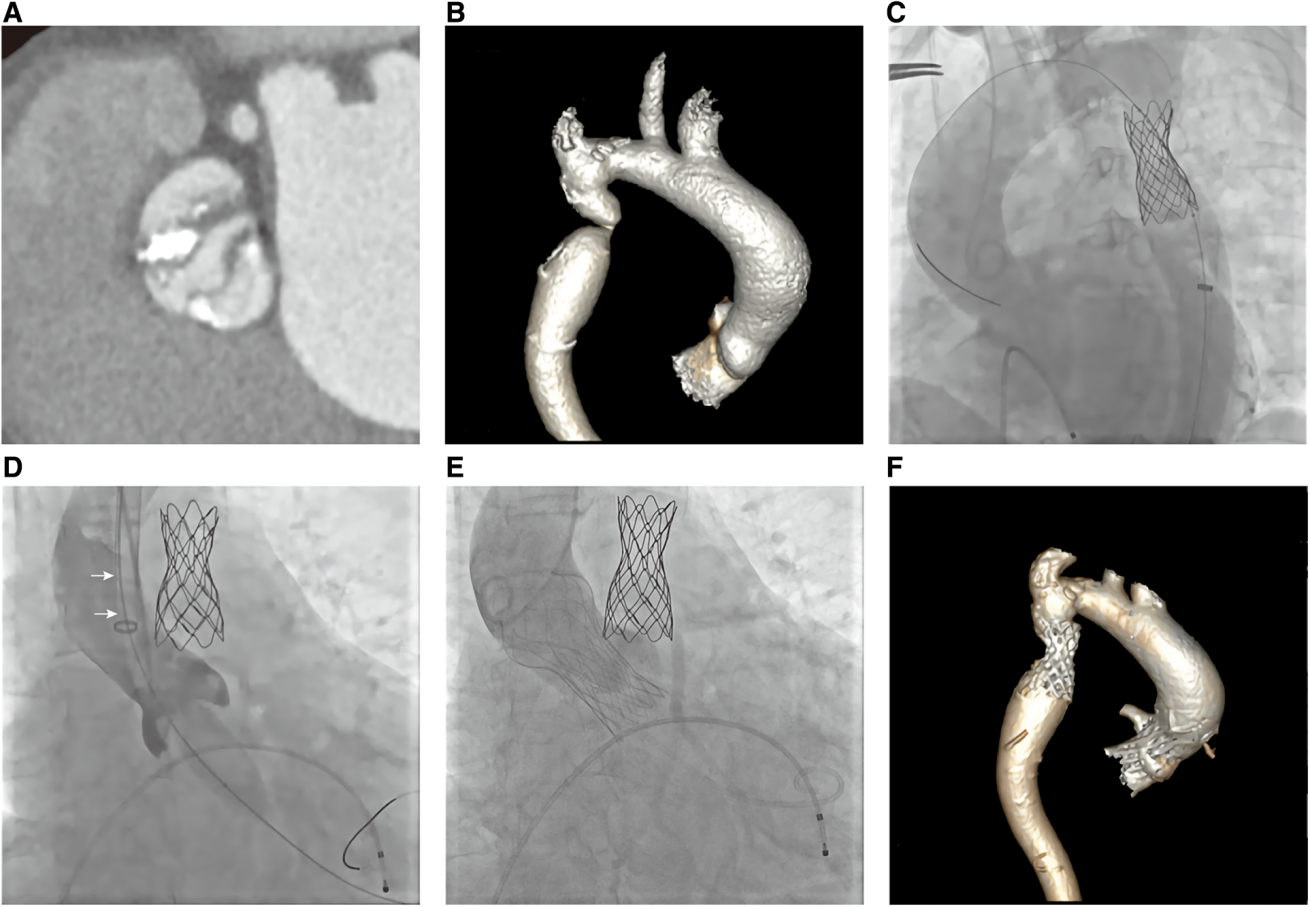
Ct aortogram and invasive aortic angiography. (**A**, **B**) CTA showed severe calcified bicuspid aortic valve stenosis (type 0) and stenotic calcified eccentric aortic coarctation 20 mm distal to the left subclavian artery; (**C**)A CP stent was implanted *via* the right femoral artery; (**D**) Aortic valve balloon dilatation *via* the left carotid artery (arrow: 20F sheath); (**E**) Prosthetic valve was implanted; (**F**) CT follow-up revealed both devices were properly positioned.

The Electrocardiography (EKG) demonstrated a sinus rhythm of 80 beats per minute, a normal axis, and left ventricular hypertrophy. Echocardiogram showed severe aortic stenosis (aortic valve area, 0.2 cm^2^; mean pressure gradient, 78 mmHg; peak velocity, 5.8 m/s) and a reduced left ventricular ejection fraction of 35% (Figure 1A). Computed tomography (CT) angiography of aorta imaging evaluation revealed severe stenotic calcified eccentric aortic coarctation located 20 mm distal to the LSA. The diameter of the narrowest part was approximately 3 mm with significant thoracic collaterals. The descending aorta is observed to be significantly twisted and angled due to eccentric stenosis. Severe asymmetric diffuse leaflet calcifications were observed in bicuspid aortic valve (BAV) type 0, as well as marked calcifications of the ascending aorta and aortic arch (Figures 2A,B). A pre-operative left heart catheterization revealed no evidence of coronary artery disease.

**Figure 3:**
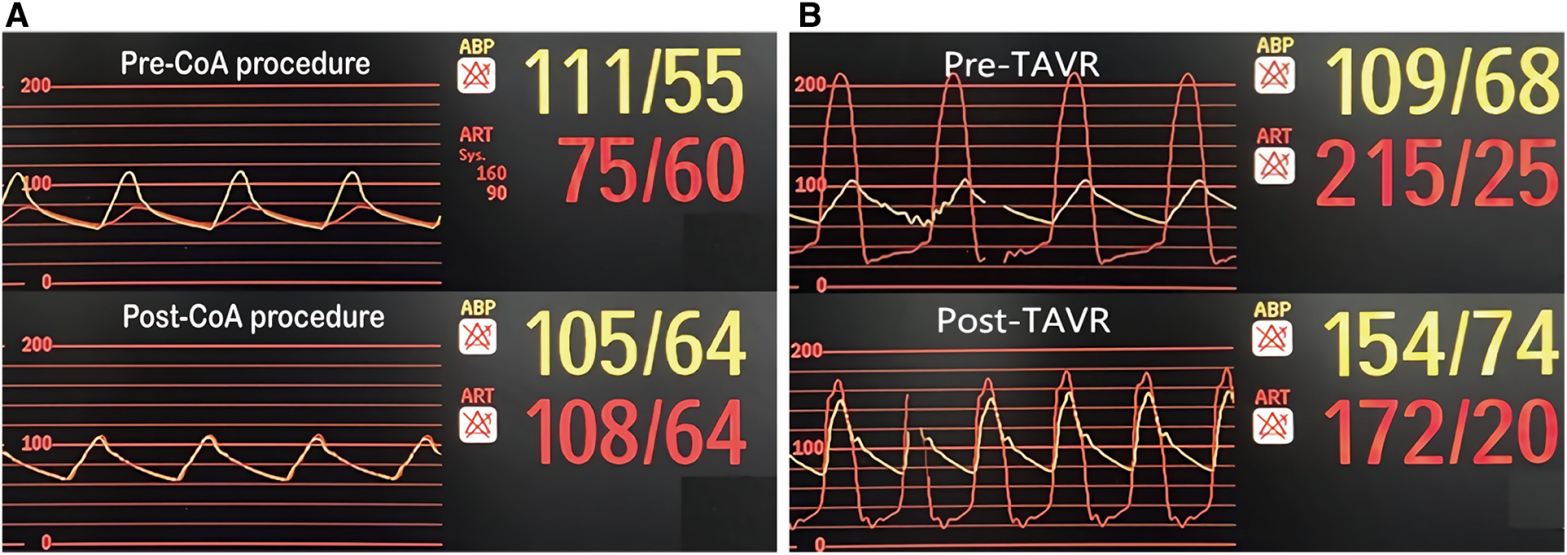
Pressure gradient variation. (**A**) Pre and post CoA interventional procedure; (**B**) Pre and post TAVR.

Based on the association of a bicuspid aortic valve and a coarctation of the aorta, the patient was diagnosed with a complex aortic coarctation and severe aortic valve stenosis. After consulting with the cardiac team and obtaining the patient's consent, we decided to perform a one-stop interventional procedure for repairing both defects. The operation was performed in the cardiac catheter lab under general anesthesia. Firstly, we implanted a 45 mm enlarged Cheatham-Platinum (CP) stent installed on a 22*45 mm BIB (balloon in balloon) catheter *via* the right femoral access, immediately distal to the LSA (Figure 2C), and achieved complete pressure gradient resolution across the CoA (from 35 mmHg to 3 mmHg) (Figure 3A). To avoid possible damage or displacement of the implanted CP stent during the delivery of the artificial valve due to the markedly twisted and angled descending aortic arch, alternate access for TAVR was considered appropriate through the left carotid artery, which was straight, wide, and free of obvious plaque.

The carotid incision was sutured with purse string to prevent bleeding. First, a 6F sheath was implanted, and the straight guide wire was inserted into the left ventricle through an Amplatze 1.0 catheter and exchanged with a 6F pigtail catheter. The 20F sheath was then replaced for balloon pre-expansion and prosthetic valve implantation. We used a 20 mm balloon to dilate the aortic valve while performing an angiographic evaluation. The findings revealed that there was no obvious balloon lumbar sign and aortic regurgitation, and that the left and right coronary arteries were well-developed (Figure 2D). A 24 mm prosthetic valve (Vitaflow Aortic Valve System, Shanghai Microport medical group) was smoothly implanted through the left carotid artery. Due to the concise and direct approach, the feedback of delivery manipulation is excellent, and the final valve deployment was in the optimal position (Figure 2E). The aortic transvalvular pressure difference decreased from 100mmHg pre-TAVR to 18 mmHg post-TAVR (Figures 1B, 3B). Trans-esophageal echocardiography (TEE) revealed no significant aortic regurgitation and auscultation indicated a markedly reduced murmur (Figures 1C, D; Supplementary Video S1). The patient was discharged on postoperative day 9. At 11 months follow-up, the patient was asymptomatic with (Table 1) well-controlled blood pressure, and echocardiography showed a well-seated prosthetic valve with a mean gradient of 7 mmHg and no valve leakage, as well as no gradient across the coarctation stent. A radiological follow-up revealed that both devices were correctly positioned (Figure 2F).

**Table 1:** Timeline from the onset of symptoms in the patient to discharge.

Timeline	Symptom onset to discharge
2 August 2021	Developed exertional chest pain and dyspnea
8 April 2022	Developed paroxysmal nocturnal dyspnea
15 April 2022	Hospitalization
18–19 April 2022	Aortic CT angiography and echocardiography
22 April 2022	One-stop interventional procedure
1 May 2022	Discharged

## Discussion

CoA is defined as a discrete constriction of the aortic lumen, typically occurring at the ductus arteriosus distal insertion site to the LSA. The pathophysiology of CoA is unclear, but it is revealed to occur in 5%–8% of patients with congenital heart disease, including two thirds of patients with BAV, while isolated CoA occurs in <0.05% of live births (3, 7). When one of these lesions is found, screening for other coexisting congenital heart abnormalities is necessary.

The natural history of unrepaired coarctation of the aorta includes the development of systemic hypertension and subsequent morbidity and death from cardiovascular disease. Long-term survivors are at risk for aortic and mitral valve dysfunction, aortic aneurysm, aortic dissection, endocarditis, systemic hypertension, heart failure, and sudden death (8). The combination of symptomatic severe aortic stenosis and CoA in more than 60-year-old patients is a rare clinical entity (9, 10). Progressive aortic valve dysfunction in patients with CoA place patients at increased risk for morbidity and mortality. However, there are no specific guidelines for adult patients concurrent with symptomatic severe aortic stenosis and CoA.

The surgical techniques described separately for each abnormality can be combined in such complex surgeries. Different techniques have been used: either Aortic valve replacement and surgical correction of the coarctation in one single step, or a staged approach (11, 12). Although surgery is still the main treatment for these diseases, it is not suitable for high-risk surgical patient.

Transcatheter intervention is the treatment of choice for single aortic disease because of good results and the less invasive nature of this technique. It is associated with similar short and mid-term hemodynamic improvements and fewer acute complications than surgery (13). However, transcatheter intervention for patients with severe aortic stenosis complicated with CoA simultaneously is rarely reported.

Our patient herein reported having CoA with multiple comorbidities, a poorly functioning heart, and a severe aortic stenosis, for which transcatheter intervention was indicated. We propose a few factors to be considered before the interventional procedure:

(1) Patients must be thoroughly evaluated before operation, including cardiac function, ascending and descending aortic and aortic root width, aortic coarctation shape, vascular access, etc. It is important to implement a cardiac team including interventional cardiologists, radiologists, and cardiac surgeons devoted to meticulously assessing these patients for optimal treatment.
(2) Access to interventions. Because CoA often leads to significant tortuosity of descending aorta, the TAVR delivery system is difficult to pass through. If the CoA is treated first with a CP stent, even if the stent improves the aortic tortuosity, it must still risk high resistance and CP stent displacement when passing through the TAVR delivery system. Therefore, different pathways for TAVR and CoA interventional therapy should be selected. In this case, the left/right carotid artery is the best choice of access for TAVR. The carotid artery with a diameter greater than 6 mm without obvious stenosis (greater than 50%), heavy artery calcification, or tortuosity is generally considered appropriate for TAVR. Furthermore, the operation time following 20F sheath implantation should be as short as possible to reduce the risk of vascular hemorrhage and cerebral ischemia. Our team completed it within 18 min.
(3) The sequence of interventional therapy for aortic stenosis and coarctation. It is difficult to predict the hemodynamic effect of correcting an abnormality on other lesions. Our strategy is to first correct abnormalities that are less disruptive to hemodynamics, such as CoA in this case. Due to the existence of collateral circulation, the pressure difference before and after CoA was only 35 mmHg. The use of CP stents had little effect on overall hemodynamics. The severe aortic stenosis which had a great impact on hemodynamics (transvalvular pressure gradient of 100 mmHg) was then treated. However, if a patient has an acute decompensated circulatory disorder with poor response to medications, severe aortic stenosis with a greater impact on hemodynamics is the key to the disease. At this time, it is reasonable to first treat the severe aortic stenosis with TAVR as soon as possible, followed by CoA.

## Conclusion

The simultaneous occurrence of severe aortic stenosis and CoA without dilation of ascending aorta in patients over the age of 60 is rare. The management is complex and must be individualized. Patients must be fully evaluated and prepared for operation, and the comprehensive assessment of the cardiac team can provide patients with the best treatment strategy. Our case report demonstrates the feasibility and efficacy of a one-stop interventional procedure in an adult patient with concurrent severely calcified BAV and CoA *via* two different vascular approaches. Transcatheter intervention, in contrast to traditional surgical approaches or two-stop interventional procedures, as a minimally invasive and novel method, offers a wider range of therapeutic methods for such diseases.

## Data Availability

The original contributions presented in the study are included in the article/**Supplementary material**, further inquiries can be directed to the corresponding authors.

## Supplementary material

Supplementary Table 1 Comparison of echocardiography values among this patient, normal population and AS patient.

Supplementary Video 1 Postoperative echocardiography showed no significant aortic regurgitation after TAVR.
