# Supplementary material for: One-stop interventional procedure for bicuspid aortic stenosis in a patient with coexisting aortic coarctation: a case report
Source: Front Cardiovasc Med. 2023 May 4;10:1162203. doi: 10.3389/fcvm.2023.1162203 (PMC10192622; doi:10.3389/fcvm.2023.1162203)
Supplement: Supplementary Table 1 — Comparison of echocardiography values among this patient, normal population and AS patient. [file Table1.docx]

TABLE 1. Timeline from the onset of symptoms in the patient to discharge.

| **Timeline** | **Symptom onset to discharge** |
| --- | --- |
| 2 August 2021 | Developed exertional chest pain and dyspnea |
| 8 April 2022 | Developed [paroxysmal nocturnal dyspnea](javascript:;) |
| 15 April 2022 | Hospitalization |
| 18-19 April 2022 | Aortic CT angiography and echocardiography |
| 22 April 2022 | One‐stop interventional procedure |
| 1 May 2022 | Discharged |
